# The association between family relationships and depressive symptoms among pregnant women: A network analysis

**DOI:** 10.3389/fpsyt.2022.919508

**Published:** 2022-08-22

**Authors:** Jingjing Wang, Yifei Pei, Jie Tang, Qian Chen, Chenlu He, Ying Zhang, Hao Hou, Xunbao Zhang, Wei Wang

**Affiliations:** School of Public Health, Xuzhou Medical University, Xuzhou, China

**Keywords:** family relationship, depression, network analysis, pregnant women, mental health

## Abstract

**Background:**

Depression of pregnant women has been a growing concern in recent years, and previous research has found that family relationships are strongly associated with depression. From a network perspective, family relationships and depression can be conceptualized as the result of interactions between individual symptoms. This research approach can elucidate the structure and mechanisms of the relationship between individual symptoms within the two groups.

**Methods:**

A total of 990 participants were recruited from the obstetrics outpatient clinic of Maternal and Child Health Hospital in Huai'an through a randomized whole-group sampling. Respondents' depressive symptoms and family relationships were self-reported using questionnaire, and the structure of the family relationship-depressive symptoms network and related centrality indicators were examined for this sample.

**Results:**

The results of the network analysis suggested that the most influential symptoms in the network of family relationship-depressive symptoms were worry, feeling worthless, equal status with husband and couple relationship. And equal status with husband was the most prominent bridging symptoms in this study. The whole network was robust in both stability and accuracy tests.

**Limitations:**

Information was obtained from subjects' self-reports, which may be subject to information bias. As a cross-sectional study, no causal link between family relationships and depressive symptoms can be established.

**Conclusion:**

Worry, feeling worthless, equal status with husband and couple relationship are central symptoms of the family relationship-depressive symptoms network structure in pregnant women. Timely and systematic multilevel interventions targeting the central symptoms may be effective in alleviating the onset of depressive symptoms in women during this period.

## Introduction

Depression is a serious human ailment that is responsible for more 'years lost' to incapacity than any other ailment worldwide (1). Based on data from the World Health Organization (2016), depression accounts for a full 10% of the total global burden of non-fatal diseases (2). Depression is characterized by a variety of symptoms, such as low emotion, self-reproach, and feeling worthless (3–5). And the prevalence of depression is steadily increasing worldwide, a meta-analysis reported that the rate of depression climbed consistently from 1982 to 2015 at an average yearly probability of 0.2%, achieving an overall prevalence of 27.2% (6).

Pregnancy period is a period of profound change for women and their spouses, and it often brings enormous difficulties and stress (7). Perinatal depression (PD) is a term that encompasses major and minor depressive episodes that occur either during pregnancy or within the first 12 months after delivery (8). A study of 17,544 people in Pakistan found that the prevalence rate of PD was as high as 30–37% (9). And the prevalence of perinatal depression in China also reached 16.3% (10). Depression in pregnant women has been shown to have a tremendous impact on the mother, child, and other family members (11–13), and it is one of the main causes of suicide and self-harm (14–17). An important factor which influences the prevalence of depression in pregnant women is family relationships (18, 19). Some studies have shown that satisfied with marriage, rapport with in-laws, and access to husband's help and support are protective factors against depression during the relationship with family members in pregnant women (18, 20, 21). Therefore, the connection between family relationships and depression among pregnant women must be considered.

In general, most previous researches on depression had always taken depression measures as a whole and computed composite scores to determine whether or not a person is depressed and how severe their depression is (22, 23). However, it fails to uncover substantial connections between particular symptoms that may be more relevant for the emergence or maintenance of co-morbid experiences like depression (24, 25). Network models can make up for this deficiency to some extent by using a web of interacting symptoms to map specific relationships between individual symptoms of a disease (26, 27). Network analysis has been extensively employed in psychopathology in recent years to understand and display patterns associated with mental diseases (24, 27, 28). In network theory, core symptoms are more likely to trigger other symptoms, and so are believed to have a significant role in initiating the start and/or maintenance of the illness (29). Therefore, identifying the central symptoms of family relationships and depression can provide a meaningful basis for reducing family relationships that trigger maternal depression.

To date no one has studied the relationship between family relationships and depressive symptoms in pregnant women using a network analysis approach. Therefore, the aim of this study was to characterize the network structure of family relationships and depressive symptoms during pregnancy of women. It's critical to pinpoint the most significant symptoms of the network model of family relationships and depressive symptoms among pregnant women.

## Methods and materials

### Settings and participants

This study used a randomized whole group sampling method and was conducted from July to December 2017. Pregnant women with first pregnancy in the second trimester and the third trimester stages who underwent obstetric outpatient checkups at Maternal and Child Health Hospital in Huai'an were selected as survey subjects. Inclusion criteria: those who underwent prenatal examination at Maternal and Child Health Hospital; willing to participate in this study and signed an informed consent form. Exclusion criteria: suffering from psychiatric and other psychotic disorders; having serious physical diseases; having underdeveloped intelligence; refusing to participate. A total of 1,000 questionnaires were distributed, and 990 complete and valid questionnaires were returned, with a return rate of 99.0%.

This study was approved by the ethics committees of Huai'an Maternal and Child Health Hospital and Xuzhou Medical University. The procedures used followed the principles of the Declaration of Helsinki.

### Measures

#### Demographic characteristics

Basic demographic characteristics were collected by questionnaire including age (*in years*), marital status (*marriage, remarried or others*), education level (*primary and below, junior high school, high school or university and above*), occupation (*workers and farmers, civil service, faculty and medical services, business services, professional and technical posts or others)*, monthly family income (< *3,000, 3,000*~*4,900, 5,000*~*7,000, or* >*7,000 yuan*), residence (*urban or rural*), resident population (*living with husband, living with husband and children, living with husband and husband's parents and living with husband and own parents or others*), only child (*yes or no*).

#### Family relationships

Family relationships were measured using a self-administered scale with 7 items, including the presence of domestic violence, equality of status with the husband, marital satisfaction, and receive support and comfort from the husband, the relationship between husband and wife, relationship with in-laws, and relationship with parents. Each entry had two options, with a score of “0” for “no/unsatisfied” and “1” for “yes/satisfied.” Among them, the presence of domestic violence is a reverse scoring question. The Cronbach's α of the scale was 0.752.

#### Depressive symptoms

Depressive symptoms of pregnant women were measured by the depression section of the Chinese Adult Mental Health Inventory (30). This scale includes the investigation of a total of 10 mental health problems such as sensitivity to interpersonal relationships, worry, maladjustment, and anxiety. The depression section measured thoughts of mood, hope, fatigue, self-reproach, interest, self-worth, sadness, and meaninglessness of life in the past 10 days. There were 8 items with 5 options for each item, ranging from “never” to “always,” with scores ranging from “1” to “5,” and a mean score of ≥2 for each item was considered to have depression disorder. The Cronbach's α of the scale was 0.851, suggesting good reliability.

### Analytical strategies

In this study, the basic demographic indicators of the study participants were first analyzed descriptively using SPSS (version 25.0) to provide an overview of samples. Next, chi-square analyses comparing different demographic indicators were performed between the depressed and non-depressed groups, with the significance level set as 0.05. Finally, the network analysis was performed in terms of network estimation, network stability, and network comparison.

### Network estimation

We use R (version 4.1.0) to perform network analysis. According to the network analysis method, each question is considered as nodes, and the pairwise correlation pairwise relations between these nodes are considered as edges (31, 32). To estimate symptom networks that account for the relationship between pregnant and postpartum women family relationships and depressive symptoms, we conducted paired Pearson correlation analyses. The network structure was estimated using the Enhanced Least Absolute Shrinkage and Selection (33, 34). We use the “qgraph” and “bootnet” packages in R for data visualization and analysis in order to obtain visual network graphs (35, 36). The algorithm uses the penalty parameter to obtain sparsity and chooses the optimal set of neighboring factors for each node (symptom) using the Extended Bayes Information Criterion (EBIC) (i.e., goodness-of-fit measure) (34, 37). When that each node is attached to multiple other nodes through edges of different weights, the final automatically constructed network is obtained, with the edge thickness and length representing the strength of the direct association between the nodes. In the network graph, the edge color represents the direction of association, a blue edge indicates a positive association between two symptoms and a red edge indicates a negative association (36). Symptom nodes that are more strongly and frequently associated with other nodes are closer to other points in the graph and more concentrated in the network.

Based on the characteristics of the network, network analysis provides quantitative centrality metrics for each node. In network analysis, network centrality metrics includes Strength, Betweenness, and Closeness. Centrality index was expressed as standardized values (z-cores). However, it has been shown that Closeness and Betweenness are not reliable in network analyses in mental health (38); therefore, only the most commonly used centrality indicator: Strength was used in this study. Strength is the sum of the weights between a particular symptom and all others directly associated symptoms (39).

### Estimation of network accuracy and stability

In this study, three methods were used to check the accuracy and stability of the network model in order to assess the robustness of the network analysis results. Firstly, the case dropped bootstrap method was used to estimate the stability of node attributes. The network is considered stable if most of the samples are excluded from the data set and the centrality index of the nodes is not observed to change significantly. The stability is represented graphically and quantitatively by the calculation of the correlation stability coefficient (CS-C) (3, 35, 40). The CS-C indicates the largest proportion of the sample that can be reduced. In general, the CS-C should not be smaller than 0.25 and preferably larger than 0.5 (35).

Secondly, bootstrapped difference tests were applied to estimate discrepancies in network properties (35). This test used the “bootnet” and “qgraph” package in R for the analysis. It relies on the 95% CI to identify whether there is a difference between two edge weights or two node centrality indices (35).

Finally, a non-parametric bootstrap method was deployed to calculate the confidence interval (CI) to estimate the accuracy of the edge weights (28, 41). The observations in the data are then randomly resampled to produce a new data set from which a 95% CI is calculated. Larger CI indicates lower accuracy of edge estimation and narrower CI indicates higher reliability of the network (31, 35).

## Result

### Study sample

A total of 990 pregnant women were included in this study, 18.2% (*n* = 180) of the participants were considered to have depression disorder. Among all participants, 70.7% lived in the cities and 29.3% in rural areas. The average age of the respondents were 28.26 years (SD = 4.86 years). Respondents living in rural areas were more likely to be depressed than those living in urban areas (χ^2^ = 6.678, *P* = 0.010). The specific socio-demographic indicators of the respondents are detailed in Table 1, and the mean scores of family relationships and depressive symptoms are detailed in Supplementary Table 1.

**Table 1:** Comparing depression across demographic indicators.

**Varible**	***n* (%)**	**Depression**		***X^2^/t***	***P***
		**Yes (%)**	**No (%)**		
Age (mean±SD)	28.26 ± 4.86	28.00 ± 4.53	28.32 ± 4.93	−0.801	0.423
Marital status					
Marriage	937 (94.6)	170 (18.1)	767 (81.9)	0.256	0.944
Remarried	45 (4.6)	8 (17.8)	37 (82.2)		
Others	8 (0.8)	2 (25.0)	6 (75.0)		
Education level					
Primary and below	13 (1.3)	3 (21.3)	10 (79.6)	0.260	0.969
Junior high school	147 (14.8)	27 (18.4)	120 (81.6)		
High school	183 (18.5)	34 (18.6)	149 (81.4)		
University and above	647 (65.4)	116 (17.9)	531 (82.1)		
Occupation					
Workers and farmers	219 (22.1)	21 (17.6)	98 (82.4)	2.618	0.625
Civil Service, Faculty and Medical Services	291 (29.4)	48 (16.5)	243 (83.5)		
Business Services	184 (18.6)	40 (21.7)	144 (78.3)		
Professional and technical posts	62 (6.3)	13 (21.0)	49 (79.0)		
Others	334 (33.7)	58 (17.4)	276 (82.6)		
Monthly family income					
<3,000 yuan	82 (8.3)	20 (24.4)	62 (75.6)	2.695	0.442
3,000~4,900 yuan	291 (29.4)	49 (16.8)	242 (83.2)		
5,000~7,000 yuan	244 (24.6)	46 (18.9)	198 (81.1)		
>7000 yuan	373 (37.7)	65 (17.4)	308 (82.6)		
Residence					
Urban	700 (70.7)	113 (16.1)	587 (83.9)	6.678	0.010
Rural	290 (29.3)	67 (23.1)	223 (76.9)		
Resident population					
Living with husband	282 (28.5)	49 (17.4)	233 (82.6)	3.109	0.543
Living with husband and children	187 (18.9)	28 (15.0)	159 (85.0)		
Living with husband and husband's parents	408 (41.2)	79 (19.4)	329 (80.6)		
Living with husband and own parents	82 (8.3)	16 (19.5)	66 (80.5)		
Others	31 (3.1)	8 (25.8)	23 (74.2)		
Only child					
Yes	261 (26.4)	42 (16.1)	219 (83.9)	1.041	0.308
No	729 (73.6)	138 (18.9)	591 (81.1)		

### Network structure and centrality measure analysis

**Figure 1:**
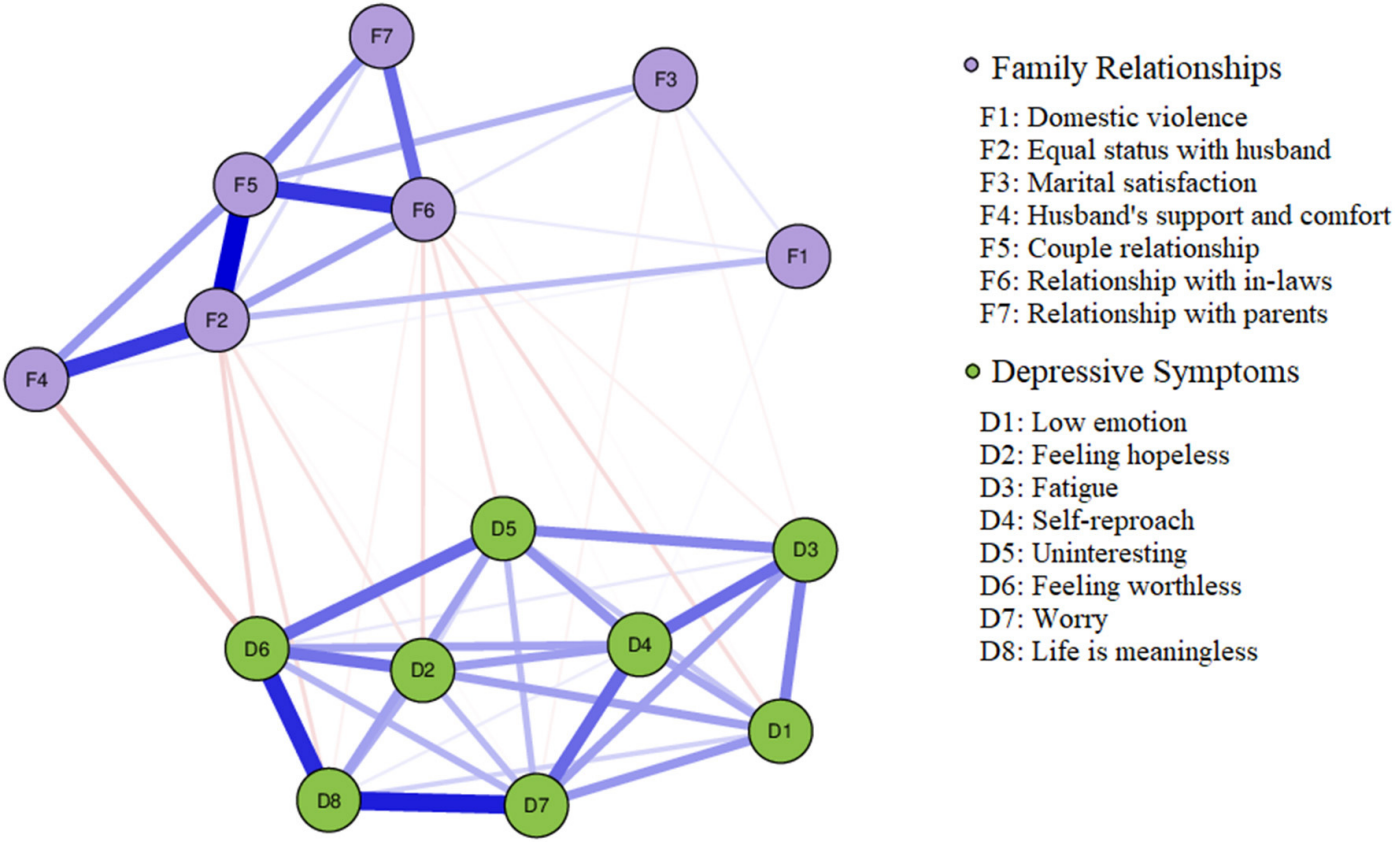
Network model estimation of family relationships and depressive symptoms among pregnant women (*N* = 990). In the diagram, symptom nodes with strong associations are relatively close to each other. The purple nodes indicate family relationship items; the green nodes indicate depressive symptom items. The dark blue line represents positive correlations. The red line represents negative correlations. The edge thickness represents the strength of association between symptom nodes.

The family relationship and depressive symptoms network was estimated with an EBICglasso model, which is shown in Figure 1. A weighted adjacency matrix was used to examine the numerical interactions between these symptoms (Supplementary Table 2). In the depression network section, the relationship between each depressive symptom and the other symptoms showed positive correlations. Of which, the node D7 (Worry) strongest associated with D6 (Feeling worthless) and D8 (Life is meaningless). In the family relationship section, F2 (Equal status with husband) was most closely related to the other points in the network. F2 (Equal status with husband) showed strong positive correlations with F5 (Couple Relationship), F4 (Husband's support and comfort). F5 (Couple Relationship) is equally closely related to other points in the network. For example, F6 (Relationship with in-laws), F4 (Husband's support and comfort), F7 (Relationship with parents) all have strong positive correlation with F5 (Couple Relationship). Overall, Family relationships were negatively associated with depressive symptoms. F4 (Husband's support and comfort) has a significant negative correlation with D6 (Feeling worthless). F6 (Relationship with in-laws) showed a distinct negative correlation with D2 (Feeling hopeless), D5 (Uninteresting) and D1 (Low Emotion).

**Figure 2:**
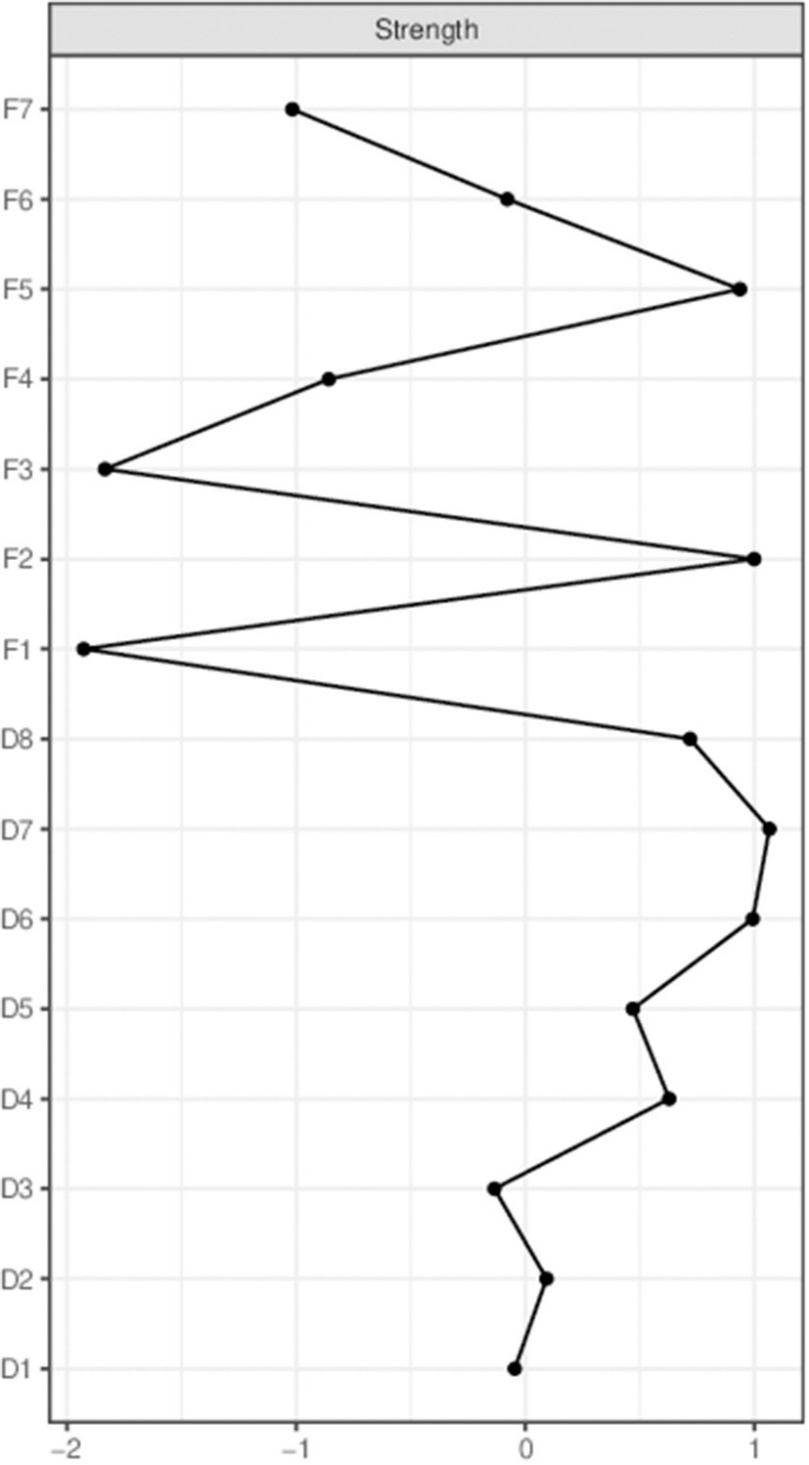
Centrality measures of all symptoms within the network. Family relationships and depressive symptoms centrality index, expressed as a standardized value z-score.

**Figure 3:**
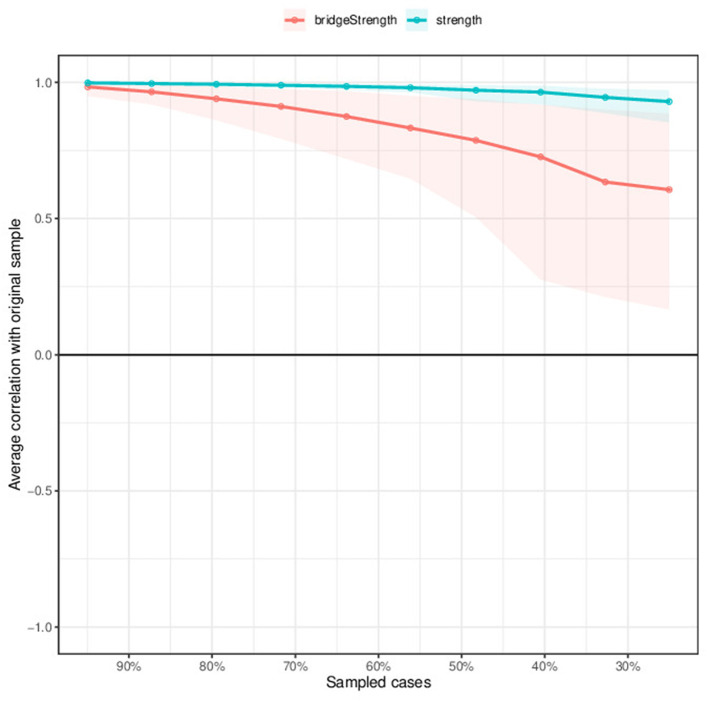
Stability of centrality indices by case dropping subset bootstrap. The x-axis represents the percentage of cases of the original sample used at each step. The y-axis represents the average of correlations between the centrality indices from the original network and the centrality indices from the networks that were re-estimated after excluding increasing percentages of cases.

Figure 2 illustrated centrality measures of all the symptoms within the network. Node D7 (Worry) is most influential in the network, followed by F2 (Equal status with husband), D6 (Feeling worthless), and F5 (Couple Relationship). In contrast, the influence of F1 (Domestic violence), F3 (Marital Satisfaction), etc. be very slight. We also did a stability analysis of the network and found it to be very stable (CS-coefficient = 0.749), demonstrating that 74.9% of the samples could be eliminated without significant changes in the network structure (Figure 3).

### Network accuracy and stability

**Figure 4:**
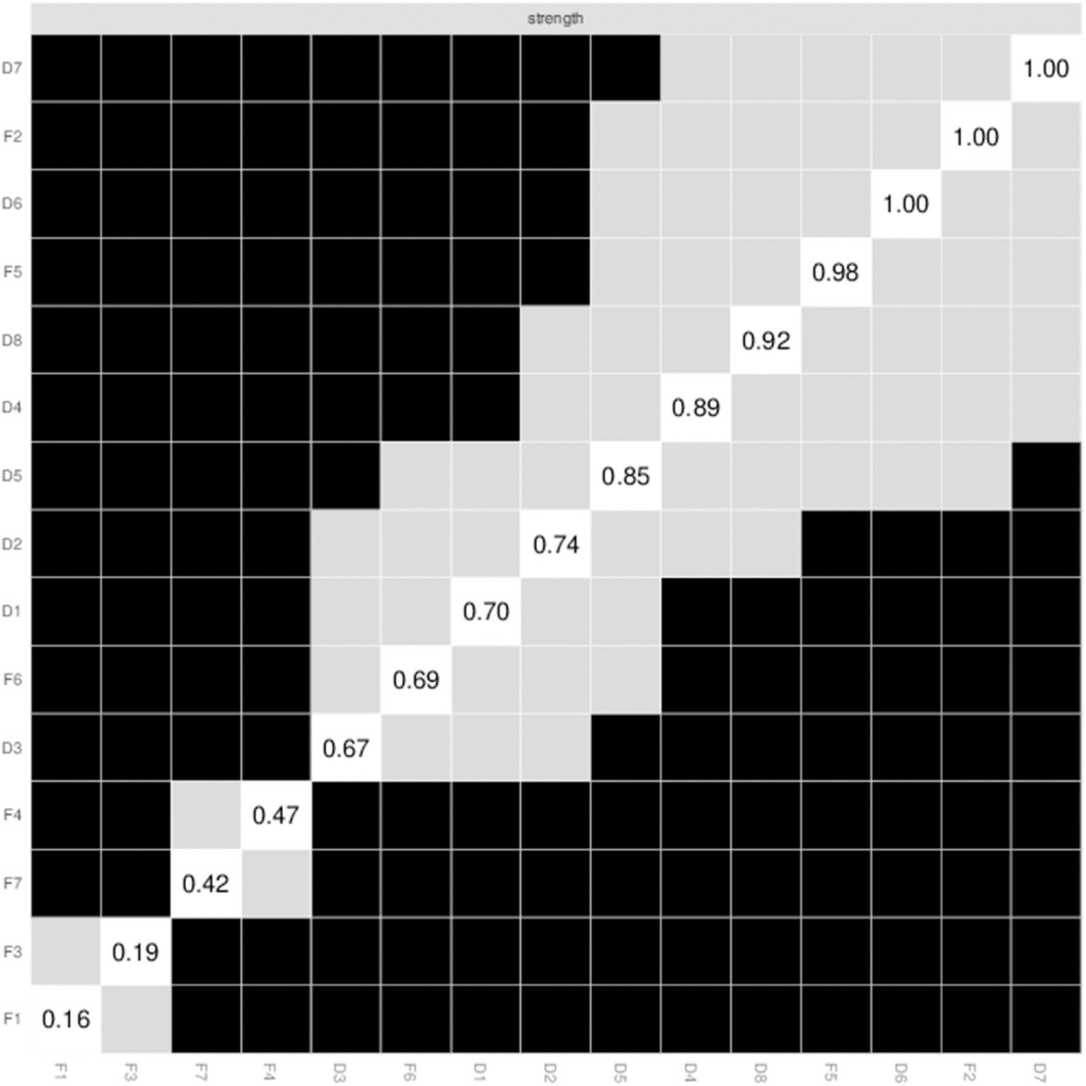
Non-parametric bootstrapped difference test for strength. Gray boxes indicate no difference between nodes, whereas black boxes indicate significant difference (α = 0.05). Values reported in the diagonal represent the strength values of each node.

The present sample's edge weights, particularly those with greater weights, were consistent with the bootstrapped sample, indicating that the existing network structure was stable (Supplementary Figure 1). Bootstrap difference tests showed that most of the comparisons between the edge weight values were statistically significant (Figure 4). Bootstrapped 95% CI for the estimated edge weights indicated that the network model was both reliable and stable (Supplementary Figure 2).

### Bridge symptoms of family relationships and depressive symptoms

**Figure 5:**
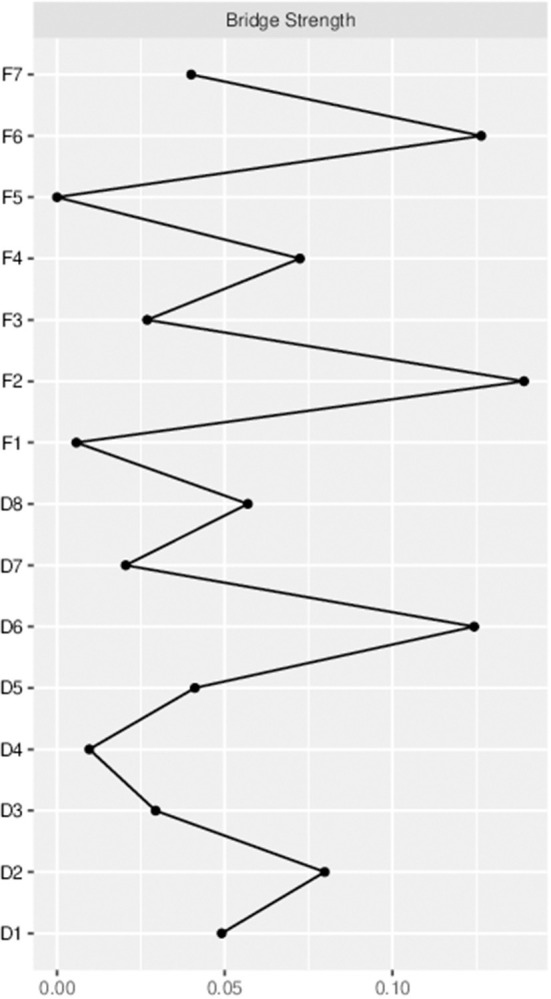
Bridge centrality indices of the family relationships and depressive symptoms among pregnant women.

Based on previous research findings, bridge strength is the best index in identifying nodes that, if deactivated, would prevent activation spread from one disorder to another (26, 42). F2 (Equal status with husband), F6 (Relationship with in-laws) and D6 (Feeling worthless) were the most prominent bridging symptoms in this study (Figure 5).

## Discussion

This study is the first to use a network analysis to explore the relationship between family relationships and depressive symptoms among Chinese pregnant women. The results of the analysis showed that in the relational network of family relationships and depressive symptoms, family relationships were generally negatively associated with depressive symptoms, that is, the more harmonious the family relationship, the less likely the mother was to experience depressive symptoms. And D7 (Worry), D6 (Feeling worthless), F2 (Equal status with husband), F5 (Couple Relationship) were the most influential nodes in the family relationship-depressive symptoms network structure. Namely, these are the symptoms most likely to trigger or maintain family relationships and depressive symptoms. In addition, the bridging symptoms connecting family relationships and depressive symptoms in this sample were F2 (Equal status with husband), F6 (Relationship with in-laws) and D6 (Feeling worthless).

In this study, 18.2% of the participants were judged to have a depressive disorder. Of which, worry is in the middle of the depressive symptom network. In previous studies of different populations, worry, although rarely in the middle, but it has a strong influence in the symptom network in this study (24, 43, 44). Some reasons can be used to explain our results. Pregnancy period are a special time for women, during that the levels of steroid and peptide hormones in pregnant women are substantial (45–47). In turn, changes in these hormones alter the hypothalamic pituitary adrenal (HPA) and hypothalamic pituitary gonadal (HPG) axes in pregnant women, and dysregulation of these endocrine axes is associated with increased maternal mood sensitivity and mood swings (45). Otherwise, multiple studies have illustrated that during period, women may have significant concerns about some problems, such as their own health and that of their child and their ability to be a competent mother (48–51). Additionally, this emotional dysregulation may exacerbate feelings of maternal worry.

The equality of status with the husband and the couple relationship play very important roles as the central node of the family relationships and the whole network of family relationships and depressive symptoms of pregnant women. In this network model, the couple relationship, although rarely directly related to depressive symptoms among pregnant women, however, it can have an impact on depressive symptoms by affecting the equality of status with the husband and relationship with in-laws. Previous studies have also found that bad couple relationship is a significant risk factor for maternal depression (52, 53), and that depression of pregnant women has a huge impact on not only themselves but also family relationships as a result of a two-way effect (54). It is not difficult to find from the results of this study that a husband's support and comfort to his wife and the equality of status with the husband are closely related to the couple's relationship. This is consistent with the results of published studies (55). When a woman is pregnant or recovering from childbirth, she will face many psychological and physical problems (56), and most pregnant women will become dependent on their husbands, whom then play a very important role in their lives (55). A harmonious couple relationship, giving the wife and husband equal status in the family and appropriate support and understanding from the husband will bring great comfort to the wife and prevent the appearance of depressive symptoms.

This study also exist some limitations. First, family relationships and depressive symptoms were measured *via* self-reported responses on questionnaire among pregnant women. This survey method may be subject to recall bias. Second, the causal relationship between family relationships and depressive symptoms could not be determined due to the non-experimental, cross-sectional study design. Future longitudinal studies are needed to assess family relationship-depressive symptom patterns. Third, given the focus of the study, our population of depressive symptoms was not patients with diagnosed depression. Therefore, the results of the network analysis in pregnant women may not be appropriate to generalize to a sample of depressed patients.

In conclusion, this network analysis revealed that the most predominant depressive symptoms that emerged during pregnancy period are worry, feeling worthless and life is meaningless. The most important family relationships are equal status with the husbands and couple relationship. And the equal status with the husbands is very significant node in the whole network model. Therefore, when women have a high family status and a good relationship with their spouse, it may help to moderate the relationship between pregnant women and other family members and reduce the occurrence of depressive symptoms.

## Data availability statement

The raw data supporting the conclusions of this article will be made available by the authors, without undue reservation.

## Ethics statement

The studies involving human participants were reviewed and approved by the Ethics Committee of Huai'an Maternal and Child Health Hospital and Xuzhou Medical University. The patients/participants provided their written informed consent to participate in this study.

## Author contributions

WW and XZ were involved in conceptualization and methodology. JW participated in data curation and writing—original draft preparation. JT, YP, QC, CH, YZ, and HH supervised and validated the study. WW participated in writing—reviewing and editing. All authors contributed to the article and approved the submitted version.

## Funding

This work was supported by the National Natural Science Foundation of China [82003484] and the Natural Science Fund for Colleges and Universities in Jiangsu Province [20KJB330005].

## Conflict of interest

The authors declare that the research was conducted in the absence of any commercial or financial relationships that could be construed as a potential conflict of interest.

## Publisher's note

All claims expressed in this article are solely those of the authors and do not necessarily represent those of their affiliated organizations, or those of the publisher, the editors and the reviewers. Any product that may be evaluated in this article, or claim that may be made by its manufacturer, is not guaranteed or endorsed by the publisher.
