# Supplementary material for: The association between family relationships and depressive symptoms among pregnant women: A network analysis
Source: Front Psychiatry. 2022 Aug 22;13:919508. doi: 10.3389/fpsyt.2022.919508 (PMC9441886; doi:10.3389/fpsyt.2022.919508)
Supplement: Supplementary file 1 [file Data_Sheet_1.docx]

Supplementary Material

**Supplementary figure and table legends**

Supplementary Table 1: Means, standard deviations, maxima, minima, skewness and kurtosis of family relationships and depressive symptoms among pregnant women.

Supplementary Table 2: Weighted adjacency matrix

Supplementary Figure 1: Bootstrapped confidence intervals of edge weights

Supplementary Figure 2: Estimation of edge weight difference by bootstrapped difference test

**Supplementary Table 1: Means, standard deviations, maxima, minima, skewness and kurtosis of maternal family relationships and depressive symptoms.**

| variable | M | SD | Min | Max | Skewness | Kurtosis |
| --- | --- | --- | --- | --- | --- | --- |
| Family relationship |  |  |  |  |  |  |
| Domestic violence | 1.97 | 0.157 | 1 | 2 | -6.061 | 34.808 |
| Equal status with husband | 1.81 | 0.392 | 1 | 2 | -1.592 | 0.536 |
| Marital Satisfaction | 1.97 | 0.169 | 1 | 2 | -5.591 | 29.322 |
| Husband's support and comfort | 1.86 | 0.344 | 1 | 2 | -2.110 | 2.457 |
| Couple Relationship | 1.93 | 0.258 | 1 | 2 | -3.325 | 9.073 |
| Relationship with in-laws | 1.84 | 0.369 | 1 | 2 | -1.831 | 1.356 |
| Relationship with parents | 1.97 | 0.180 | 1 | 2 | -5.207 | 25.167 |
| Depression symptom |  |  |  |  |  |  |
| Low Emotion | 1.65 | 0.698 | 1 | 5 | 0.825 | 0.571 |
| Feeling hopeless | 1.37 | 0.621 | 1 | 5 | 1.875 | 4.712 |
| Fatigue | 1.88 | 0.760 | 1 | 5 | 0.834 | 1.173 |
| Self-reproach | 1.63 | 0.739 | 1 | 5 | 1.039 | 0.871 |
| Uninteresting | 1.51 | 0.721 | 1 | 5 | 1.502 | 2.409 |
| Feeling worthless | 1.35 | 0.630 | 1 | 5 | 2.151 | 5.890 |
| Worry | 1.47 | 0.672 | 1 | 5 | 1.685 | 4.255 |
| Life is meaningless | 1.34 | 0.633 | 1 | 5 | 2.216 | 5.860 |

**Supplementary Table 2: Weighted adjacency matrix**

|  | F1 | F2 | F3 | F4 | F5 | F6 | F7 | D1 | D2 | D3 | D4 | D5 | D6 | D7 | D8 |
| --- | --- | --- | --- | --- | --- | --- | --- | --- | --- | --- | --- | --- | --- | --- | --- |
| F1 | 0 | 0.088 | 0.028 | 0.013 | 0.025 | 0 | 0 | 0 | 0 | 0 | 0.006 | 0 | 0 | 0 | 0 |
| F2 | 0.088 | 0 | 0.034 | 0.249 | 0.324 | 0.120 | 0.045 | -0.004 | -0.036 | 0 | 0 | 0 | -0.052 | 0 | -0.042 |
| F3 | 0.028 | 0.034 | 0 | 0 | 0.097 | 0 | 0 | 0 | 0 | -0.013 | 0 | 0 | 0 | -0.014 | 0 |
| F4 | 0.013 | 0.249 | 0 | 0 | 0.132 | 0 | 0 | 0 | 0 | 0 | 0 | 0 | -0.073 | 0 | 0 |
| F5 | 0.025 | 0.324 | 0.097 | 0.132 | 0 | 0.255 | 0.148 | 0 | 0 | 0 | 0 | 0 | 0 | 0 | 0 |
| F6 | 0 | 0.120 | 0 | 0 | 0.255 | 0 | 0.188 | -0.042 | -0.044 | -0.017 | -0.004 | -0.005 | 0 | -0.001 | -0.015 |
| F7 | 0 | 0.045 | 0 | 0 | 0.148 | 0.188 | 0 | -0.004 | 0 | 0 | 0 | -0.036 | 0 | 0 | 0 |
| D1 | 0 | -0.004 | 0 | 0 | 0 | -0.041 | -0.004 | 0 | 0.120 | 0.159 | 0.111 | 0.073 | 0 | 0.130 | 0.057 |
| D2 | 0 | -0.036 | 0 | 0 | 0 | -0.044 | 0 | 0.120 | 0 | 0 | 0.123 | 0.048 | 0.176 | 0.088 | 0.104 |
| D3 | 0 | 0 | -0.013 | 0 | 0 | -0.017 | 0 | 0.159 | 0 | 0 | 0.184 | 0.153 | 0.027 | 0.121 | 0 |
| D4 | 0.006 | 0 | 0 | 0 | 0 | -0.004 | 0 | 0.111 | 0.123 | 0.184 | 0 | 0.137 | 0.116 | 0.189 | 0.023 |
| D5 | 0 | 0 | 0 | 0 | 0 | -0.005 | -0.036 | 0.073 | 0.048 | 0.153 | 0.137 | 0 | 0.187 | 0.087 | 0.120 |
| D6 | 0 | -0.052 | 0 | -0.072 | 0 | 0 | 0 | 0 | 0.176 | 0.027 | 0.116 | 0.187 | 0 | 0.095 | 0.271 |
| D7 | 0 | -0.006 | -0.014 | 0 | 0 | -0.001 | 0 | 0.130 | 0.088 | 0.121 | 0.189 | 0.087 | 0.095 | 0 | 0.287 |
| D8 | 0 | -0.042 | 0 | 0 | 0 | -0.015 | 0 | 0.057 | 0.104 | 0 | 0.023 | 0.120 | 0.271 | 0.287 | 0 |

Weighted adjacency matrix based on factors to represent the weight of direct edges between nodes.


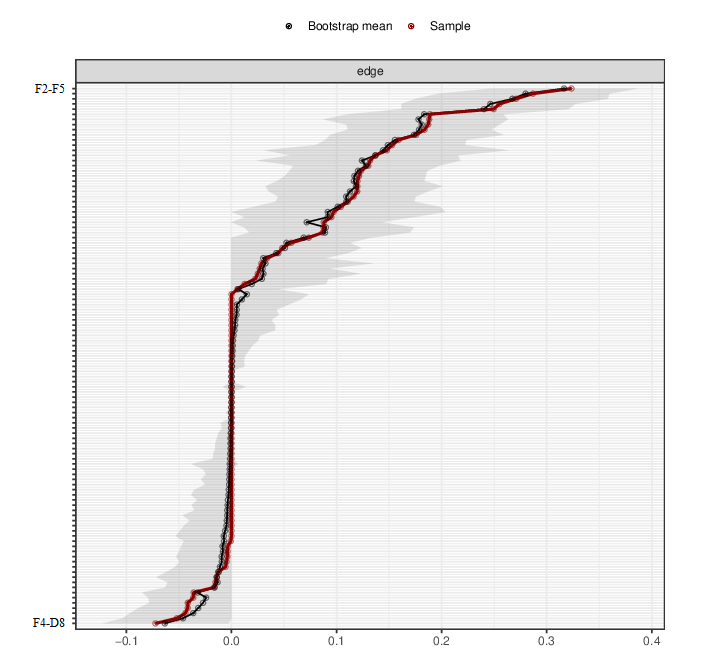


**Supplementary Figure 1: Bootstrapped confidence intervals of edge weights.** The black dots indicate the values of each edge weight, ordered from the highest to the lowest value. The gray area represents the 95% Confidence Intervals of edge weights, estimated with the non-parametric bootstrap procedure (bootnet package). Wide intervals indicate lower stability and narrow intervals indicate higher stability. Because the image vertical coordinates are too dense, only the first and last two coordinates are marked in this paper.


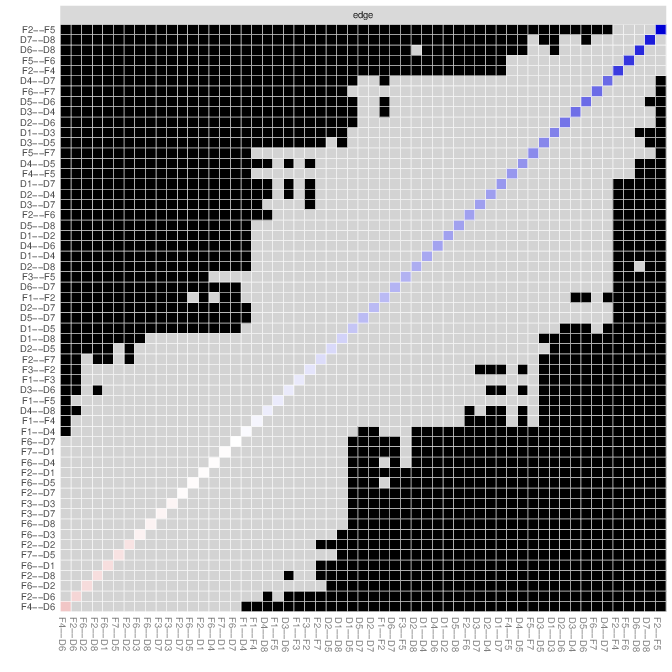


**Supplementary Figure 2: Estimation of edge weight difference by bootstrapped difference test.** Bootstrapped difference tests between edge weights in the network. Gray boxes indicate edges that do not significantly differ from one-another. Black boxes represent edges with significant difference from one another (α = 0.05). Blue boxes in the edge-weight plot indicate positive correlations.
